# Association between red blood cell distribution width and long-term mortality in acute respiratory failure patients

**DOI:** 10.1038/s41598-020-78321-2

**Published:** 2020-12-03

**Authors:** Wei Zhang, Yadan Wang, Jun Wang, Shaochun Wang

**Affiliations:** 1 Department of Respiratory and Critical Care Medicine, Shaanxi Provincial People’s Hospital, Xi’an, China; 2 Ruibiao (Wuhan) Biotechnology Co. Ltd, Wuhan, China

**Keywords:** Biomarkers, Diseases

## Abstract

The red cell distribution width (RDW) has been reported to be positively correlated with short-term mortality of pulmonary disease in adults. However, it is not clear whether RDW was associated with the long-term prognosis for acute respiratory failure (ARF). Thus, an analysis was conducted to evaluate the association between RDW and 3-year mortality of patients by the Cox regression analysis, generalized additives models, subgroup analysis and Kaplan–Meier analysis. A total of 2999 patients who were first admitted to hospital with ARF were extracted from the Medical Information Mart for Intensive Care III database (MIMIC-III). The Cox regression analysis showed that the high RDW was associated with 3-year mortality (HR 1.10, 95% CI 1.07, 1.12, P < 0.0001) after adjusting for age, gender, ethnicity and even co-morbid conditions. The ROC curve illustrated the AUC of RDW was 0.651 (95% CI 0.631, 0.670) for prediction of 3-year mortality. Therefore, there is an association between the RDW and survival time of 3 years follow-up, particularly a high RDW on admission was associated with an increased risk of long-term mortality in patients with ARF. RDW may provide an alternative indicator to predict the prognosis and disease progression and more it is easy to get.

## Introduction

Acute respiratory failure (ARF) is one of the most common complications in critically ill patients admitted to the intensive care unit (ICU) and usually leads to high mortality^1^. Even with mechanical ventilation and extracorporeal membrane oxygenation (ECMO), approximately 36% of patients died consequently during hospitalization^2^. Although there are some scoring systems used to predict the clinical outcomes of patients in ICU, no one is specifically available for patients with AFR. Several studies have demonstrated that hypoproteinemia, malnutrition, predecease functional status, and Acute Physiology and Chronic Health Evaluation II (APACHE II) score^3,4^ are related to the need for mechanical ventilation in ARF, but most of these studies were conducted in patients with acute exacerbation of chronic obstructive pulmonary disease (AECOPD) who made up only a small proportion of patients with ARF. Delayed recognition of clinical deterioration in AFR is common, which leads to poor clinical outcomes and increased in-hospital mortality^5^. Therefore, it is necessary to find useful indicators to predict the clinical outcomes of ARF.

Red blood cell distribution width (RDW) is a parameter of red blood cell volume heterogeneity obtained by a standard complete blood count^6^. It is commonly used to identify different types of anemia and reactive bone marrow states and it is also a simple, widely used, and inexpensive parameter. In recent years, some clinical studies have shown that there is a correlation between RDW and some acute diseases, such as brain infarction, sepsis, infective endocarditis and diabetic ketoacidosis^7–11^. In addition, several studies have reported the predictive effect of RDW on lung disease^12–16^, while the predictive effect of RDW on acute respiratory failure has not been reported. Therefore, our objective is to study whether RDW is useful to predict clinical outcomes of patients with ARF.

## Results

### Characteristics of patients

There were 2999 patients with ARF included in the study, with 1654 (55.15%) patients died within 3 years. We divided all the patients into three groups, according to the tertiles of RDW: the low group (RDW < 14.1%), the middle group (14.1% ≤ RDW < 15.7%) and the high group (RDW ≥ 15.7%). Patients with high RDW were more likely to have high APS III score, OASIS score and more comorbidities. They also had a high 1-year and 3-year mortality. The characteristics and hematologic laboratory data of the study participants were displayed in Table 1.

**Table 1:** Characteristics of the enrolled patients according to RDW.

	All subjects	RDW (%)			P-value
		< 14.1	≥ 14.1 < 15.7	≥ 15.7	
		N = 947	N = 1049	N = 1003	
Age (years)	64.88 ± 16.57	62.12 ± 18.34	66.28 ± 15.90	66.02 ± 15.14	< 0.001
Female	1348 (44.95%)	412 (43.51%)	456 (43.47%)	480 (47.86%)	0.076
**Ethnicity**					
Caucasian	2072 (69.09%)	649 (68.53%)	747 (71.21%)	676 (67.40%)	0.005
Black	208 (6.94%)	61 (6.44%)	52 (4.96%)	95 (9.47%)	
Asian	71 (2.37%)	20 (2.11%)	25 (2.38%)	26 (2.59%)	
Others	648 (21.61%)	217 (22.91%)	225 (21.45%)	206 (20.54%)	
**Admission type**					
Emergency	2728 (90.96%)	881 (93.03%)	937 (89.32%)	910 (90.73%)	0.016
Urgent	179 (5.97%)	44 (4.65%)	80 (7.63%)	55 (5.48%)	
Elective	92 (3.07%)	22 (2.32%)	32 (3.05%)	38 (3.79%)	
**Comorbidity**					
Pulmonary circulatory disease	255 (8.50%)	65 (6.86%)	88 (8.39%)	102 (10.17%)	0.032
Chronic pulmonary disease	810 (27.01%)	223 (23.55%)	325 (30.98%)	262 (26.12%)	< 0.001
Renal failure	356 (11.87%)	48 (5.07%)	121 (11.53%)	187 (18.64%)	< 0.001
Liver disease	305 (10.17%)	34 (3.59%)	79 (7.53%)	192 (19.14%)	< 0.001
Metastatic cancer	191 (6.37%)	44 (4.65%)	58 (5.53%)	89 (8.87%)	< 0.001
Congestive heart failure	1064 (35.48%)	266 (28.09%)	396 (37.75%)	402 (40.08%)	< 0.001
SID30	11.31 ± 8.32	8.8 ± 7.6	10.9 ± 8.1	14.1 ± 8.4	< 0.001
**Mechanical ventilation**					
Yes	2649 (88.33%)	856 (90.39%)	940 (89.61%)	853 (85.04%)	< 0.001
No	350 (11.67%)	91 (9.61%)	109 (10.39%)	150 (14.96%)	
**Severity scale**					
APS III	54.54 ± 23.35	48.94 ± 21.70	52.72 ± 22.33	61.74 ± 24.10	< 0.001
OASIS	38.60 ± 8.08	37.77 ± 7.69	38.61 ± 7.90	39.36 ± 8.56	< 0.001
qSOFA	1.89 ± 0.68	1.85 ± 0.69	1.92 ± 0.67	1.89 ± 0.69	0.113
SIRS	3.14 ± 0.89	3.16 ± 0.91	3.14 ± 0.87	3.12 ± 0.88	0.501
**Type of respiratory failure**					
Hypoxic respiratory failure	2373 (79.13%)	762 (80.46%)	806 (76.84%)	805 (80.26%)	0.077
Hypercarbic respiratory failure	626 (20.87%)	185 (19.54%)	243 (23.16%)	198 (19.74%)	0.062
Time in ICU (days)	10.36 ± 10.01	10.43 ± 10.61	10.42 ± 10.04	10.24 ± 9.38	0.892
Time in hospital (days)	17.10 ± 16.37	16.19 ± 15.23	17.21 ± 15.63	17.86 ± 18.05	0.076
1-year mortality	1443 (48.12%)	331 (34.95%)	454 (43.28%)	658 (65.60%)	< 0.001
3-year mortality	1654 (55.15%)	398 (42.03%)	530 (50.52%)	726 (72.38%)	< 0.001

*RDW* red cell distribution width, *SID30* Elixhauser Comorbidity Index, *APS III* Acute Physiology Score III, *OASIS* Oxford acute severity of illness score, *qSOFA* quick sequential organ failure assessment score, *SIRS* systemic inflammatory response syndrome.

### Association of RDW levels and clinical outcomes

The univariate Cox regression analysis (Table S2) indicated that age, gender, APS III score, OASIS score, qSOFA score, SIRS score, SID30, mechanical ventilation, congestive heart failure, chronic renal failure, liver disease, and metastatic cancer were significantly associated with 3‑year all-cause mortality. The above variables also were associated with 1-year mortality (Table S3).

**Figure 1:**
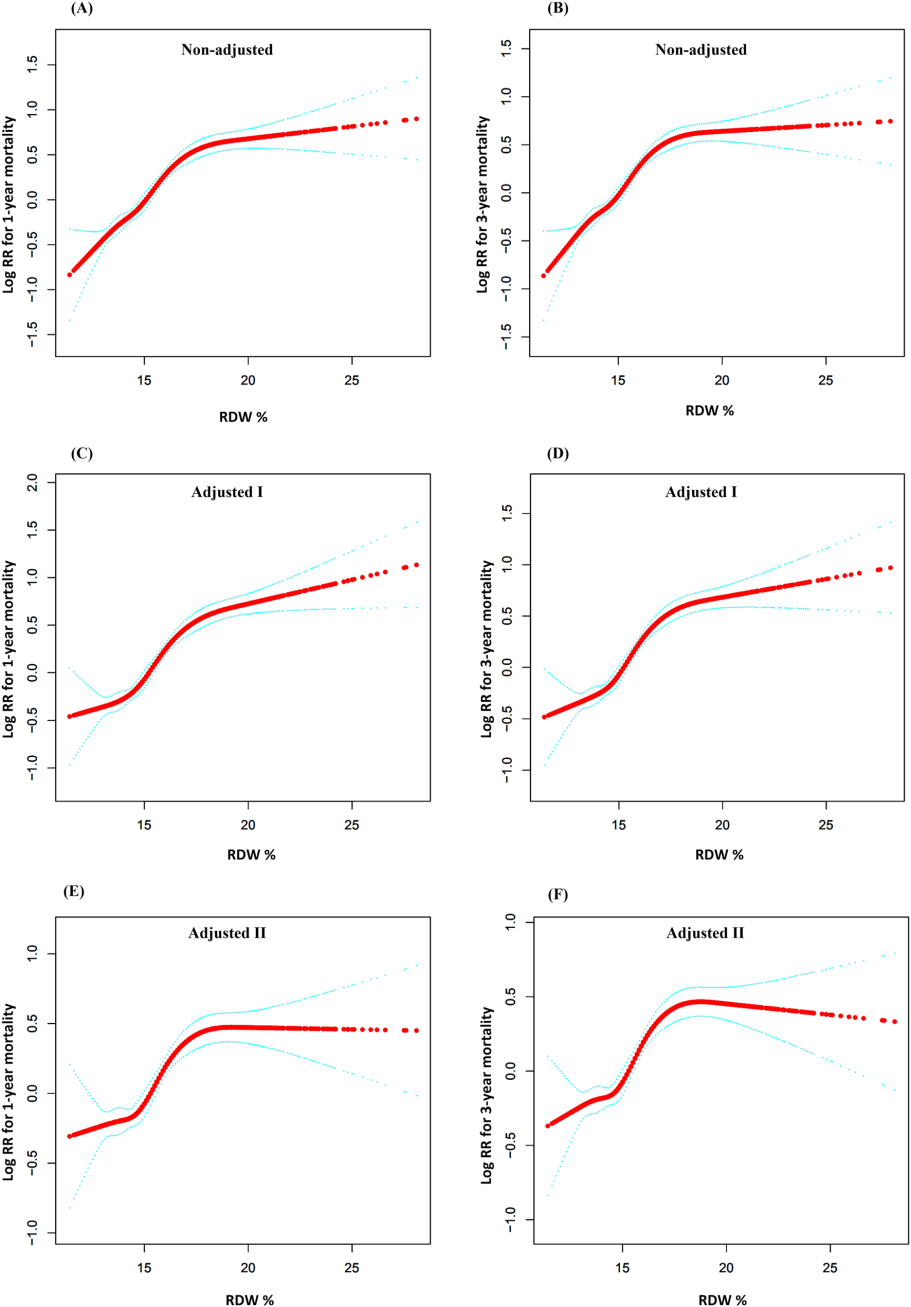
Association between RDW and clinical outcomes for patients with ARF in different multivariable models. (**A**,**C**,**E**) association between RDW and 1-year mortality for patients with ARF. (**B**,**D**,**F**) association between RDW and 3-year mortality for patients with ARF. Adjusted I for age, gender and ethnicity; Adjusted II for age, gender, ethnicity, liver disease, metastatic cancer, congestive heart failure, renal failure, APS III score, SID30, OASIS, qSOFA, SIRS and mechanical ventilation. *HR* indicates hazard risk, *ARF* acute respiratory failure, *RDW* red cell distribution width, *SID30* Elixhauser Comorbidity Index, *APS III* acute physiology score III, *OASIS* Oxford acute severity of illness score, *qSOFA* quick sequential organ failure assessment score, *SIRS* Systemic inflammatory response syndrome.

Figure 1 presents the association between baseline RDW levels and risk of 1-year and 3-year mortality (log HR). Taking RDW on admission as a continuous variable, there was a significant positive association with the clinical outcomes in different multivariable models. We further evaluated this finding by the multivariable Cox regression analysis, which was shown in Table 2. As a continuous variable, an SD increase in RDW levels was associated with a 10% higher risk of 3-year mortality in the adjusted model (HR 1.10, 95% CI 1.07, 1.12, P < 0.0001), and there was a similar trend for 1-year mortality (HR 1.10, 95% CI 1.07, 1.12, P < 0.0001). When RDW was assessed as tertiles, there was a significant high risk of 3-year mortality (HR 1.73, 95% CI 1.52, 1.98, P < 0.0001) and 1-year mortality (HR 1.74, 95% CI 1.51, 2.00, P < 0.0001) in patients in high group compared with patients in low group.

**Table 2:** Association of RDW with 1-year mortality and 3-year mortality.

RDW, %	Non-adjusted		Adjust I		Adjust II	
	HR (95% CI)	P-value	HR (95% CI)	P-value	HR (95% CI)	P-value
**1-year mortality**						
RDW Per 1 sd	1.15 (1.13, 1.18)	< 0.0001	1.16 (1.14, 1.18)	< 0.0001	1.10 (1.07, 1.12)	< 0.0001
RDW tertile						
T1	1.0 (Ref)		1.0 (Ref)		1.0 (Ref)	
T2	1.28 (1.11, 1.47)	0.0006	1.19 (1.03, 1.37)	0.0187	1.11 (0.96, 1.28)	0.1534
T3	2.36 (2.07, 2.70)	< 0.0001	2.22 (1.95, 2.54)	< 0.0001	1.74 (1.51, 2.00)	< 0.0001
**3-year mortality**						
RDW Per 1 sd	1.15 (1.13, 1.17)	< 0.0001	1.15 (1.13, 1.17)	< 0.0001	1.10 (1.07, 1.12)	< 0.0001
RDW tertile						
T1	1.0 (Ref)		1.0 (Ref)		1.0 (Ref)	
T2	1.26 (1.11, 1.44)	0.0004	1.17 (1.03, 1.33)	0.0194	1.11 (0.97, 1.26)	0.1237
T3	2.30 (2.03, 2.60)	< 0.0001	2.15 (1.90, 2.44)	< 0.0001	1.73 (1.52, 1.98)	< 0.0001

Adjusted I for age, gender and ethnicity.

Adjusted II for age, gender, ethnicity, liver disease, metastatic cancer, congestive heart failure, renal failure, APS III score, SID30, OASIS, qSOFA, SIRS and mechanical ventilation.

*HR* indicates hazard risk, *CI* confidence interval.

Subgroup analyses were performed according to age, sex, ethnicity, pulmonary circulatory disease, chronic pulmonary disease, renal failure, liver disease, metastatic cancer, congestive heart failure and mechanical ventilation for the primary and secondary outcomes. The association between RDW on admission and the clinical outcomes was similar for all strata (Tables 3, S4).

**Table 3:** Subgroup analysis of the associations between RDW and 3-year all-cause mortality by multivariable Cox regression.

	No. of patients	HR (95% CI)	P-value
**Gender**			
Male	1651	1.13 (1.10, 1.17)	< 0.0001
Female	1348	1.07 (1.04, 1.10)	< 0.0001
**Age (years)**			
< 65	1433	1.13 (1.10, 1.17)	< 0.0001
≥ 65	1566	1.07 (1.04, 1.10)	< 0.0001
**Ethnicity**			
Caucasian	2072	1.10 (1.07, 1.13)	< 0.0001
Black	208	1.10 (1.01, 1.19)	0.0202
Asian	71	1.08 (0.90, 1.29)	0.4193
Others	648	1.08 (1.03, 1.13)	0.0018
**Congestive heart failure**			
No	1935	1.09 (1.06, 1.12)	< 0.0001
Yes	1064	1.10 (1.07, 1.14)	< 0.0001
**Pulmonary circulatory disease**			
No	2744	1.10 (1.07, 1.12)	< 0.0001
Yes	255	1.10 (1.02, 1.18)	0.0092
**Chronic pulmonary disease**			
No	2189	1.09 (1.06, 1.12)	< 0.0001
Yes	810	1.11 (1.06, 1.16)	< 0.0001
**Renal failure**			
No	2643	1.09 (1.06, 1.11)	< 0.0001
Yes	356	1.17 (1.10, 1.24)	< 0.0001
**Liver disease**			
No	2694	1.10 (1.07, 1.12)	< 0.0001
Yes	305	1.06 (1.01, 1.12)	0.0262
**Metastatic cancer**			
No	2808	1.10 (1.08, 1.13)	< 0.0001
Yes	191	1.04 (0.98, 1.11)	0.2327
**Mechanical ventilation**			
No	350	1.14 (1.08, 1.21)	< 0.0001
Yes	2649	1.09 (1.06, 1.11)	< 0.0001

Adjusted for age, gender, ethnicity, liver disease, metastatic cancer, congestive heart failure, renal failure, APS III score, SID30, OASIS, qSOFA, SIRS and mechanical ventilation, if not stratified.

*HR* indicates hazard risk, *CI* confidence interval.

### ROC curve analysis

**Figure 2:**
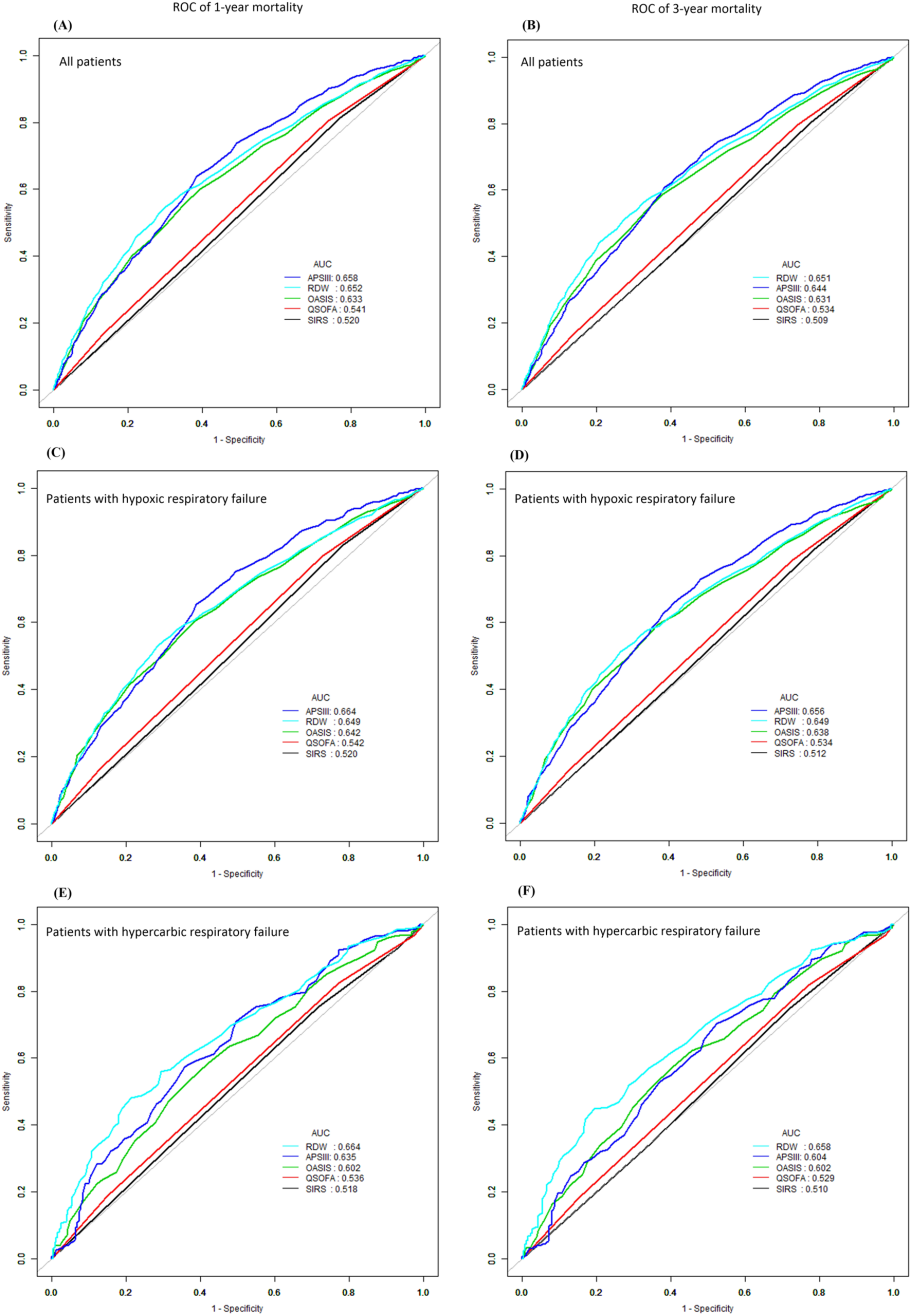
ROC analyses of predictors of red cell distribution width (RDW) for 3-year and 1-year mortality in critical ill patients, which were compared with APS III, OASIS, qSOFA and SIRS. (**A**,**B**) ROC for all patients; (**C**,**D**) ROC for patients with hypoxic respiratory failure; (**E**,**F**) ROC for patients with hypercarbic respiratory failure. *APS III* acute physiology score III, *OASIS* Oxford acute severity of illness score, *qSOFA* quick sequential organ failure assessment score, *SIRS* Systemic inflammatory response syndrome, *ROC* receiver operating curve.

ROC curves of 3-year mortality and 1-year mortality generated using the indicated variables (RDW, APS III, OASIS, qSOFA and SIRS) are plotted in Fig. 2A,B. The AUC of 3-year all-cause mortality for RDW was 0.651 (95% CI 0.631, 0.670), which was significantly higher than qSOFA and SIRS scores (P < 0.0001, Fig. 2B). And for 1-year mortality, the AUC was 0.652 (95% CI 0.632, 0.671), which is also higher than qSOFA and SIRS scores. According to the CO_2_ levels in arterial blood, respiratory failure is divided into hypoxic respiratory failure and hypercarbic respiratory failure. We further analyzed the long-term predictive value of RDW for different types of respiratory failure, and the results were consistent (Fig. 2C–F). The AUC and P value of RDW and different scoring systems for clinical outcomes were shown in Table 4.

**Table 4:** ROC of RDW and severity scales for different types of respiratory failure.

Predictor	All patients		Patients with hypoxic respiratory failure		Patients with hypercarbic respiratory failure	
	AUC	P-value	AUC	P-value	AUC	P-value
**1-year mortality**						
RDW	0.652		0.649		0.664	
APS III	0.658	0.6475	0.664	0.2748	0.635	0.3214
OASIS	0.633	0.1815	0.642	0.6393	0.602	0.0521
qSOFA	0.541	< 0.0001	0.542	< 0.0001	0.536	< 0.0001
SIRS	0.520	< 0.0001	0.520	< 0.0001	0.518	< 0.0001
**3-year mortality**						
RDW	0.651		0.649		0.658	
APS III	0.644	0.6267	0.656	0.6060	0.604	0.0811
OASIS	0.631	0.1495	0.638	0.4867	0.602	0.0848
qSOFA	0.534	< 0.0001	0.534	< 0.0001	0.529	< 0.0001
SIRS	0.509	< 0.0001	0.512	< 0.0001	0.510	< 0.0001

*ROC* receiver operating curve, *AUC* area under curve.

### Survival status of patients with different admission RDW level

**Figure 3:**
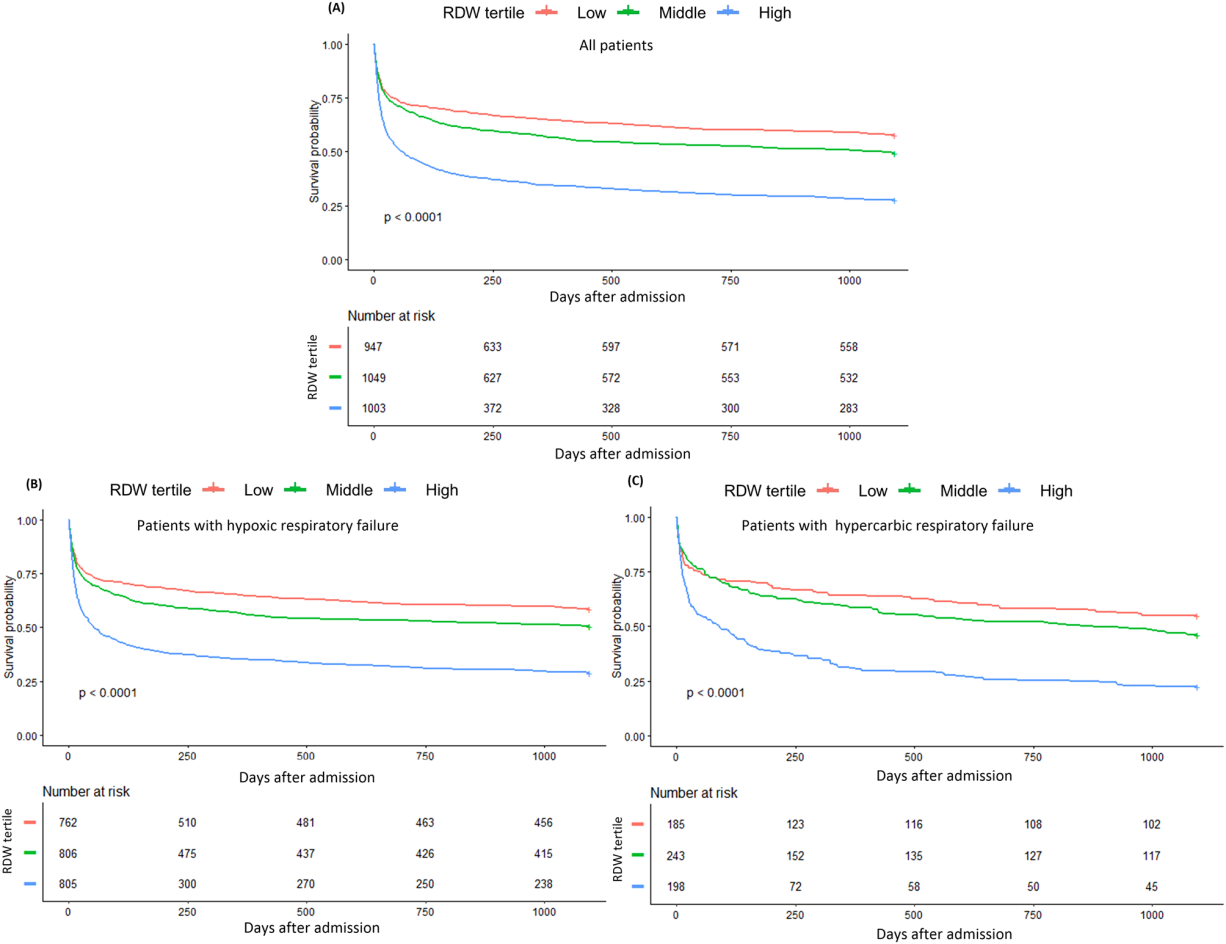
Kaplan–Meier (K-M) survival curves for 3-year all-cause mortalities. (**A**) 3-year all-cause mortalities for all patients; (**B**) 3-year all-cause mortalities for patients with hypoxic respiratory failure; (**C**) 3-year all-cause mortalities for patients with hypercarbic respiratory failure.

The K–M survival curves showed that patients in the high RDW group had significantly shorter survival time and higher mortality than patients in the middle and low RDW group (log-rank test: P < 0.001), which was presented in Fig. 3A. And for different types of respiratory failure, we obtain the same results (Fig. 3B,C).

## Discussion

Our results demonstrated that there is a significant positive correlation between RDW on admission and long-term mortality of patients with ARF. High RDW was associated with high 3-year and 1-year mortality, and this correlation was also demonstrated in all subgroup analyses. We further analyzed the relationship between the number of reticulocyte and the RDW grouping, and the results indicated that the count of reticulocyte increased gradually according to the three groups (low, medium and high) of RDW patients, with a significant difference (Table S5). The results suggest a fact that hypoxemia lead to the increase of red blood cells, thus affecting red blood cell size.

RDW represents the size variation of circulating red blood cells. Any physiologic process that affects the morphology of red blood cells and causes the early release of young cells into circulation can lead to the increase of RDW. First, ARF can lead to severe hypoxemia which induces EPO release that can reach a level of several 100-fold^17^. After the onset of ARF, hypoxia can lead to the production of hypoxia-inducible factors (HIFs) which may induced cell-type specific gene expression changes, thus, increasing the production of EPO in the renal and liver under the action of hypoxia-induced transcription factor 2 (HIF-2)^17^. EPO increases regardless of hypoxemia from a variety of causes, including COPD, pulmonary hypertension, interstitial lung disease, or heart failure^18–20^. The EPO not only increases the rate of formation of RBCs, but also increases the volume of RBCs leading to an increase in RDW^18,21^. Second, Previous studies have shown that RDW is associated with the outcomes of several inflammatory diseases, including acute pancreatitis, sepsis, and chronic heart failure^22–25^. Abnormal RDW elevation is closely related to proinflammatory factors^24,26^. Proinflammatory cytokines can decrease the half-life of red blood cells and change the morphology of red blood cells^27^. Moreover, inflammation can retard the maturation of red blood cells, causing an upregulation of reticulocytosis and the release of a large number of reticulocytes into the peripheral circulation, leading to the increase of RDW^28,29^. Third, in patients with mechanical ventilation and acute lung injury, oxidative stress presents to reactive active oxygen free radicals which can curtail the lifespan of red blood cells, thus promoting the release of young cells into the circulation^30^. Renal failures, hyperglycemia and vitamin D3 deficiency have also been suggested as factors linking RDW to poor clinical outcomes^6^.

Combined with the results of reticulocyte and previous studies, we proposed the following hypothesis: after the onset of ARF, hypoxemia can promote the synthesis of EPO in the kidney and liver, thereby increasing the rate of formation of RBCs and affecting the morphology of red blood cells. Inflammation and oxidative stress prevent the maturation of red blood cells, resulting in the release of large numbers of reticulocytes into the peripheral circulation and leading to the increase of RDW.

To our knowledge, this is the first study to demonstrate high RDW as an independent predictor for the long-term outcomes of patients with ARF. Previous studies have shown that high RDW is associated with a lower PaO2/FiO2 rate, a lower ventilation-free day and a greater need for ventilation in acute respiratory failure. Tiffany et al.^28^ found that in a cohort of 637 patients with ARF, participants with high RDW at admission had a 32% reduction in total length of 30-day period without ventilator compared with the patients with RDW in normal range (RR: 0.68; 95% CI 0.55–0.83, P < 0.001). Tom et al.^14^ showed that after adjusting for anemia, age, and disease severity (PRISM III), high RDW was an independent risk factor for mechanical ventilation of children (OR 2.6; 95% CI 1.4–4.9; P = 0.004). Danny et al.^31^ constructed survival curves after adjusting for Charlson comorbidity index and reported that 60-day readmission for AECOPD was higher in patients with high RDW than patients with normal RDW (P = 0.0038). Moreover, results from a retrospective cohort study of MIMIC-III data including 404 eligible ARDS patients suggest that RDW ≥ 14.5% was an independent predictor of 30-day (OR 1.91, 95% CI 1.08, 3.39) and 90-day mortality (OR 2.56, 95% CI 1.50, 4.37)^13^. These findings are similar to our results and high RDW is associated with poor prognosis in patients with respiratory failure.

In addition, we compared the value of RDW and other severity scale scores to predict long-term mortality. The results showed that, the AUC of RDW has a significant higher predictive value than the qSOFA and SIRS scores. In our study, only APS III had predictive accuracy comparable to RDW on long-term clinical outcomes for ARF. However, the clinical application of APS III is very limited, which requires more patients’ data to calculate, including vital signs, blood gas analysis, laboratory test results, etc. On the contrary, RDW is a routine parameter of blood routine. It can be obtained easily without any additional cost, which may make it more convenient for clinical use to early detect the patients with poor long-term prognosis, and early to take different treatment and nursing measures.

The advantage of the present study is that we analyzed the long-term prognosis of ARF which is difficult to be observed in other studies, because we had a complete follow-up database and a large sample size. However, our study has some limitations. First, as a retrospective cohort study, it is impossible to adjust for all confounders. We have adjusted for known confounders as much as possible, but there are still some unmeasured variables that may affect our results. Due to several variables were not recorded in MIMIC-III, we lacked some indicators about inflammatory response (such as C-reactive protein and interleukin 6) which may affect our results. Therefore, prospective studies are needed to confirm these results. Second, we only measured RDW levels of patients on admission; we did not investigate the trends, which could reveal more information. Third, this is a single-center study, and the results of the study should also be interpreted with caution when implicating in other populations and areas. Fourth, considering the effects of blood transfusion and anemia on RDW, patients with anemia and received blood transfusion were excluded according to the ICD9 code of MIMIC database. Therefore, our conclusions could not interpret these patients.

## Conclusion

In conclusion, our data suggested an association between the RDW and survival time of 3-year follow-up, particularly a high RDW on admission was associated with an increased risk of long-term mortality in patients with ARF. RDW may provide an alternative indicator to predict the prognosis and disease progression and more it is easy to get. There are several theoretical mechanisms to explain the increase of RDW, and further studies are needed to validate the relationship between EPO, pro-inflammatory factors, reticulocytes, and RDW in the serum of patients with ARF.

## Patients and methods

### Data sources

This is a retrospective cohort study with the data of patients obtained from the Medical Information Mart for Intensive Care III (MIMIC-III). We gained access to the database by taking an online course at the National Institutes of Health and passing the 'protection Human Research Participants’ exam (no. 6182750). MIMIC III is a single-center and freely accessible database which contains 53,423 adult patients (aged over 16) during 2001 to 2012 in the ICU of the Beth Israel Deaconess Medical Center in Boston^32^. The establishment of the MIMIC III database was approved by the institutional review board of Beth Israel deacons Medical Center and Massachusetts Institute of Technology. Informed consent was not required because all protected health information has been de-identified.

### Patients

**Figure 4:**
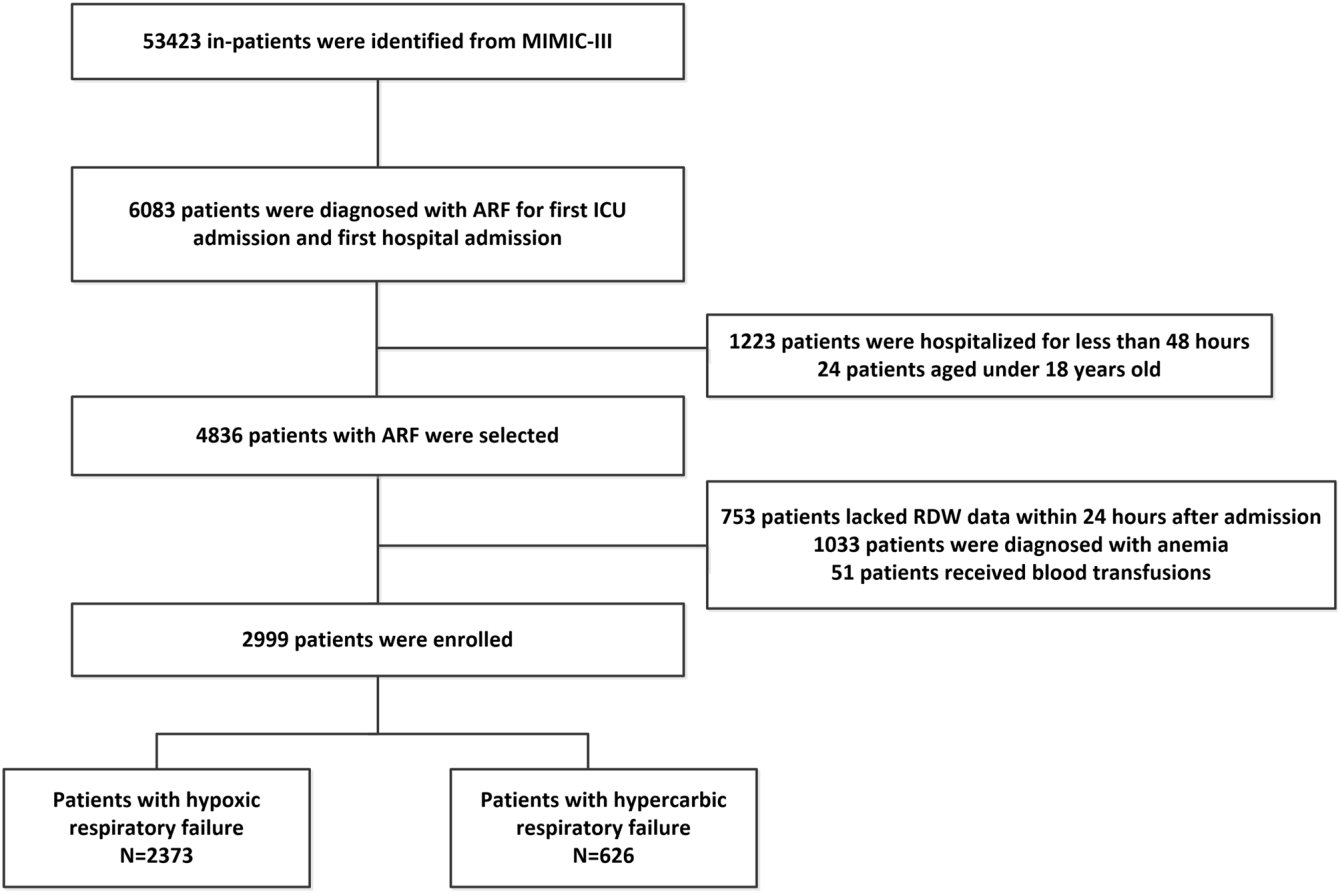
Flow chart of the current study. *ARF* acute respiratory failure, *RDW* red cell distribution width, *ICU* intensive care unit.

All the patients who were over the age of 18 and hospitalized in ICU for more than 48 h were included in the study. Patients who did not have an RDW data within 24 h after admission were excluded. Patients with anemia or received blood transfusion were also excluded. If a patient was admitted repeatedly during the study period, we used only the record of his first hospital admission (Fig. 4).

### Variables and outcome measures

The following variables were extracted or calculated: age, sex, comorbidities, ethnicity, admission type, 1-year and 3-year mortality, length of ICU and hospital stay, RDW (within 24hour of ICU admission), mechanical ventilation, Acute Physiology Score III (APS III), the Oxford Acute Severity of Illness Score (OASIS), the quick Sequential Organ Failure Assessment score (qSOFA), the Systemic Inflammatory Response Syndrome score (SIRS) and the Elixhauser Comorbidity Index (SID30). The APS III, OASIS, qSOFA and SIRS scores were estimated for all patients within 24 h of ICU admission. The comorbidities were identified and extracted by ICD-9 code in MIMIC-III database. Detailed ICD-9 codes and disease names of each comorbidities are presented in S1. The codes of Structured Query Language which was used to extract the data were obtained from https://github.com/MIT-LCP/mimic-website^32^. The primary outcome was the 3-year mortality following hospital admission, and the secondary outcome was 1-year mortality.

### Statistical analysis

The whole participants were divided into three groups according to the tertiles of RDW. The categorical variables were presented as percentages and the continuous variables were expressed as the mean (SD) or IQR. The baseline characteristics and the scoring systems were compared by chi-square test when they were categorical variables and by Kruskal–Wallis test when they were continuous variables for the three groups. Multivariable Cox regression model and generalized linear models with a logit link were used to test the independent effects of RDW on 3-year and 1-year all-cause mortality with crude and full model. The adjusting variables included age, gender, ethnicity, liver disease, metastatic cancer, congestive heart failure, renal failure, APS III score, ECI (SID30), OASIS, qSOFA, SIRS and mechanical ventilation. We selected those confounders based on their associations with the outcomes of interest or a change in effect estimate of more than 10%.

Additionally, a subgroup analysis was performed to determine whether there were differences of each subgroup in RDW prediction of clinical outcomes. The effects of RDW (as a continuous variable) on the risks of ARF were estimated using COX regression models among subgroups classified by adjusting for age, gender, ethnicity, liver disease, metastatic cancer, congestive heart failure, renal failure, APS III score, SID30, OASIS, qSOFA, SIRS and mechanical ventilation, if not stratified. The Kaplan–Meier (K–M) method and log-rank tests were used to compare the differences in survival rate between each group of patients with RDW at admission. We performed the Receiver operating characteristic (ROC) curves to assess the predictive value of RDW for 3-year and 1-year mortality of patients.

All the data were processed and analyzed by EmpowerStats software (www.empowerstats.com version R.3.4.3) and statistical software package R. A two-tailed P value < 0.05 was considered statistically significant.

## Supplementary Information

Supplementary information.
