# Supplementary material for: Association between red blood cell distribution width and long-term mortality in acute respiratory failure patients
Source: Sci Rep. 2020 Dec 3;10:21185. doi: 10.1038/s41598-020-78321-2 (PMC7713121; doi:10.1038/s41598-020-78321-2)
Supplement: Supplementary file 1 — Supplementary information. [file 41598_2020_78321_MOESM1_ESM.docx]

**Association between red blood cell distribution width and long-term mortality in acute respiratory failure patients**

Wei Zhang^1^, Yadan Wang^2^, Jun Wang MD^1^, Shaochun Wang, MD^1^

**S1. ICD-9 codes and definition of comorbidities**

|  | ^ICD9^ | ^Disease name^ |
| --- | --- | --- |
| ^Pulmonary circulatory disease^ | ^41511, 41512, 41513, 41519, 4160, 4161, 4162, 4168, 4169, 4179^ | ^Primary pulmonary hypertension; Kyphoscoliotic heart disease; Chronic pulmonary embolism; Chronic pulmonary heart disease; Iatrogenic pulmonary embolism and infarction; Septic pulmonary embolism; Saddle embolus of pulmonary artery; Other pulmonary embolism and infarction; Unspecified disease of pulmonary circulation;^ |
| ^Chronic pulmonary disease^ | ^490, 4910, 4911, 49120, 49121, 49122, 4918, 4919, 4920, 4928, 49300, 49301, 49302, 49310, 49311, 49312, 49320, 49321, 49322, 49381, 49382, 49390, 49391, 49392, 494, 4941, 4950, 4951, 4952, 4953, 4954, 4955, 4956, 4957, 4958, 4959, 496, 500, 501, 502, 503, 504, 505, 5064^ | ^Bronchitis, not specified as acute or chronic; Simple chronic bronchitis;^  ^Mucopurulent chronic bronchitis; Obstructive chronic bronchitis without exacerbation; Obstructive chronic bronchitis with (acute) exacerbation;^  ^Obstructive chronic bronchitis with acute bronchitis; Other chronic bronchitis; Unspecified chronic bronchitis; Emphysema; Extrinsic asthma, unspecified; Extrinsic asthma with status asthmaticus; Extrinsic asthma with (acute) exacerbation; Intrinsic asthma, unspecified; Intrinsic asthma with status asthmaticus; Intrinsic asthma with (acute) exacerbation; Chronic obstructive asthma, unspecified; Chronic obstructive asthma with status asthmaticus; Chronic obstructive asthma with (acute) exacerbation; Exercise induced bronchospasm; Cough variant asthma; Asthma, unspecified type, unspecified; Asthma, unspecified type, with status asthmaticus; Asthma, unspecified type, with (acute) exacerbation; Bronchiectasis without acute exacerbation; Bronchiectasis with acute exacerbation; Farmers' lung; Bagassosis; Bird-fanciers' lung; Suberosis; Malt workers' lung; Mushroom workers' lung; Maple bark-strippers' lung; "Ventilation" pneumonitis; Other specified allergic alveolitis and pneumonitis; Unspecified allergic alveolitis and pneumonitis; Chronic airway obstruction, not elsewhere classified; Coal workers' pneumoconiosis; Asbestosis; Pneumoconiosis due to other silica or silicates; Pneumoconiosis due to other inorganic dust; Pneumopathy due to inhalation of other dust; Pneumoconiosis, unspecified; Chronic respiratory conditions due to fumes and vapors;^ |
| ^Renal failure^ | ^5851, 5852, 5853, 5854, 5855, 5856, 5859, 586, V568, V420, V561, V562, V5631, V5632, V4511, V4512, V560^ | ^Chronic kidney disease, Stage I; Chronic kidney disease, Stage II (mild); Chronic kidney disease, Stage III (moderate); Chronic kidney disease, Stage IV (severe); Chronic kidney disease, Stage V; End stage renal disease; Chronic kidney disease, unspecified; Renal failure, unspecified; Encounter for other dialysis; Kidney replaced by transplant; Fitting and adjustment of extracorporeal dialysis catheter; Fitting and adjustment of peritoneal dialysis catheter; Encounter for adequacy testing for hemodialysis; Encounter for adequacy testing for peritoneal dialysis; Renal dialysis status; Noncompliance with renal dialysis; Encounter for extracorporeal dialysis^ |
| ^Liver disease^ | ^07022, 07023, 07032, 07044, 4560, 4561, 45620, 45621, 5710, 5712, 5713, 57140, 57141, 57142, 57149, 5715, 5716, 5718, 5719, 5723, 5728, 5735, V427^ | ^Chronic viral hepatitis B with hepatic coma without hepatitis delta; Chronic viral hepatitis B with hepatic coma with hepatitis delta; Chronic viral hepatitis B without mention of hepatic coma without mention of hepatitis delta; Chronic hepatitis C with hepatic coma; Esophageal varices with bleeding; Esophageal varices without mention of bleeding; Esophageal varices in diseases classified elsewhere, with bleeding; Esophageal varices in diseases classified elsewhere, without mention of bleeding; Alcoholic fatty liver; Alcoholic cirrhosis of liver; Alcoholic liver damage, unspecified; Chronic hepatitis, unspecified; Chronic persistent hepatitis; Autoimmune hepatitis; Other chronic hepatitis; Cirrhosis of liver without mention of alcohol; Biliary cirrhosis; Other chronic nonalcoholic liver disease; Unspecified chronic liver disease without mention of alcohol; Portal hypertension; Other sequelae of chronic liver disease; Hepatopulmonary syndrome; Liver replaced by transplant^ |
| ^Metastatic cancer^ | ^19882, 19889, 1990, 1991, 1960, 1961, 1962, 1963, 1965, 1966, 1968, 1969, 1970, 1971, 1972, 1973, 1974, 1975, 1976, 1977, 1978, 1980, 1981, 1982, 1983, 1984, 1985,1986, 1987, 19881, 20970, 20971, 20972, 20973, 20974, 20975, 20979, 78951^ | ^Secondary malignant neoplasm of genital organs; Secondary malignant neoplasm of other specified sites; Disseminated malignant neoplasm without specification of site; Other malignant neoplasm without specification of site; Secondary and unspecified malignant neoplasm of lymph nodes of head, face, and neck; Secondary and unspecified malignant neoplasm of intrathoracic lymph nodes; Secondary and unspecified malignant neoplasm of intra-abdominal lymph nodes; Secondary and unspecified malignant neoplasm of lymph nodes of axilla and upper limb; Secondary and unspecified malignant neoplasm of lymph nodes of inguinal region and lower limb; Secondary and unspecified malignant neoplasm of intrapelvic lymph nodes; Secondary and unspecified malignant neoplasm of lymph nodes of multiple sites; Secondary and unspecified malignant neoplasm of lymph nodes, site unspecified; Secondary malignant neoplasm of lung; Secondary malignant neoplasm of mediastinum; Secondary malignant neoplasm of pleura; Secondary malignant neoplasm of other respiratory organs; Secondary malignant neoplasm of small intestine including duodenum; Secondary malignant neoplasm of large intestine and rectum; Secondary malignant neoplasm of retroperitoneum and peritoneum; Malignant neoplasm of liver, secondary; Secondary malignant neoplasm of other digestive organs and spleen; Secondary malignant neoplasm of kidney; Secondary malignant neoplasm of other urinary organs; Secondary malignant neoplasm of skin; Secondary malignant neoplasm of brain and spinal cord; Secondary malignant neoplasm of other parts of nervous system; Secondary malignant neoplasm of bone and bone marrow; Secondary malignant neoplasm of ovary; Secondary malignant neoplasm of adrenal gland; Secondary malignant neoplasm of breast; Secondary neuroendocrine tumor, unspecified site; Secondary neuroendocrine tumor of distant lymph nodes; Secondary neuroendocrine tumor of liver; Secondary neuroendocrine tumor of bone; Secondary neuroendocrine tumor of peritoneum; Secondary Merkel cell carcinoma; Secondary neuroendocrine tumor of other sites; Malignant ascites^ |
| ^Congestive heart failure^ | ^39891, 4280, 4281, 42820, 42821, 42822, 42823, 42830, 42831, 42832, 42833, 42840, 42841, 42842, 42843, 4289^ | ^Rheumatic heart failure (congestive); Congestive heart failure, unspecified; Left heart failure; Systolic heart failure, unspecified; Acute systolic heart failure; Chronic systolic heart failure; Acute on chronic systolic heart failure; Diastolic heart failure, unspecified; Acute diastolic heart failure; Chronic diastolic heart failure; Acute on chronic diastolic heart failure; Combined systolic and diastolic heart failure, unspecified; Acute combined systolic and diastolic heart failure; Chronic combined systolic and diastolic heart failure; Acute on chronic combined systolic and diastolic heart failure; Heart failure, unspecified^ |

**S2. Univariate Cox regression analysis of all patients on 3-year mortality**

| Variables | HR | 95% CI | P-value |
| --- | --- | --- | --- |
| Gender |  |  |  |
| Male | 1.0 |  |  |
| Female | 1.11 | (1.01, 1.23) | 0.0277 |
| Age (years) | 1.03 | (1.02, 1.03) | <0.0001 |
| Ethnicity |  |  |  |
| Caucasian | 1.0 |  |  |
| Black | 0.81 | (0.66, 0.99) | 0.0414 |
| Asian | 0.97 | (0.71, 1.33) | 0.8688 |
| Others | 1.02 | (0.91, 1.15) | 0.7479 |
| Admission type |  |  |  |
| Emergency | 1.0 |  |  |
| Urgent | 1.34 | (1.08, 1.66) | 0.0082 |
| Elective | 1.03 | (0.74, 1.45) | 0.8631 |
| RDW | 1.15 | (1.13, 1.17) | <0.0001 |
| Mechanical ventilation |  |  |  |
| No | 1.0 |  |  |
| Yes | 0.76 | (0.66, 0.87) | <0.0001 |
| Comorbidity |  |  |  |
| Congestive heart failure |  |  |  |
| No | 1.0 |  |  |
| Yes | 1.30 | (1.18, 1.43) | <0.0001 |
| Pulmonary circulatory disease |  |  |  |
| No | 1.0 |  |  |
| Yes | 0.99 | (0.83, 1.17) | 0.9087 |
| Chronic pulmonary disease |  |  |  |
| No | 1.0 |  |  |
| Yes | 1.04 | (0.93, 1.16) | 0.4983 |
| Renal failure |  |  |  |
| No | 1.0 |  |  |
| Yes | 1.26 | (1.09, 1.45) | 0.0014 |
| Liver disease |  |  |  |
| No | 1.0 |  |  |
| Yes | 1.34 | (1.15, 1.56) | 0.0001 |
| Metastatic cancer |  |  |  |
| No | 1.0 |  |  |
| Yes | 2.56 | (2.17, 3.01) | <0.0001 |
| SID30 | 1.03 | (1.02, 1.03) | < 0.0001 |
| Severity scale |  |  |  |
| APSIII | 1.01 | (1.01, 1.02) | <0.0001 |
| OASIS | 1.04 | (1.04, 1.05) | <0.0001 |
| qSOFA | 1.16 | (1.08, 1.25) | <0.0001 |
| SIRS | 1.07 | (1.01, 1.13) | 0.0166 |

HR: indicates hazard risk; CI: confidence interval；

RDW: red cell distribution width; SID30: Elixhauser Comorbidity index; APS III: Acute Physiology Score III; OASIS: Oxford Acute Severity of Illness Score; qSOFA: quick Sequential Organ Failure Assessment score; SIRS: Systemic Inflammatory Response Syndrome.

**S3. Univariate Cox regression analysis of all patients on 1-year mortality**

| Variables | HR | 95% CI | P-value |
| --- | --- | --- | --- |
| Gender |  |  |  |
| Male | 1.0 |  |  |
| Female | 1.12 | (1.01, 1.24) | 0.0361 |
| Age (years) | 1.03 | (1.02, 1.03) | <0.0001 |
| Ethnicity |  |  |  |
| Caucasian | 1.0 |  |  |
| Black | 0.78 | (0.63, 0.97) | 0.0259 |
| Asian | 0.89 | (0.63, 1.27) | 0.5192 |
| Others | 1.05 | (0.92, 1.19) | 0.4829 |
| Admission type |  |  |  |
| Emergency | 1.0 |  |  |
| Urgent | 1.43 | (1.13, 1.81) | 0.0030 |
| Elective | 1.11 | (0.74, 1.64) | 0.6213 |
| RDW | 1.15 | (1.13, 1.18) | <0.0001 |
| Mechanical ventilation |  |  |  |
| No | 1.0 |  |  |
| Yes | 0.76 | (0.66, 0.88) | 0.0004 |
| Comorbidity |  |  |  |
| Congestive heart failure |  |  |  |
| No | 1.0 |  |  |
| Yes | 1.24 | (1.11, 1.38) | <0.0001 |
| Pulmonary circulatory disease |  |  |  |
| No | 1.0 |  |  |
| Yes | 0.93 | (0.77, 1.12) | 0.4534 |
| Chronic pulmonary disease |  |  |  |
| No | 1.0 |  |  |
| Yes | 0.97 | (0.87, 1.09) | 0.6490 |
| Renal failure |  |  |  |
| No | 1.0 |  |  |
| Yes | 1.24 | (1.07, 1.44) | 0.0041 |
| Liver disease |  |  |  |
| No | 1.0 |  |  |
| Yes | 1.46 | (1.25, 1.71) | <0.0001 |
| Metastatic cancer |  |  |  |
| No | 1.0 |  |  |
| Yes | 2.61 | (2.21, 3.08) | <0.0001 |
| SID30 | 1.03 | (1.02, 1.03) | < 0.0001 |
| Severity scale |  |  |  |
| APSIII | 1.02 | (1.01, 1.02) | <0.0001 |
| OASIS | 1.05 | (1.04, 1.05) | <0.0001 |
| qSOFA | 1.20 | (1.11, 1.30) | <0.0001 |
| SIRS | 1.10 | (1.04, 1.17) | 0.0018 |

HR: indicates hazard risk; CI: confidence interval；

RDW: red cell distribution width; SID30: Elixhauser Comorbidity index; APS III: Acute Physiology Score III; OASIS: Oxford Acute Severity of Illness Score; qSOFA: quick Sequential Organ Failure Assessment score; SIRS: Systemic Inflammatory Response Syndrome.

**S4: Subgroup analysis of the associations between RDW and 1-year all-cause mortality by multivariable Cox regression**

|  | No. of patients | HR (95%CI) P-value |
| --- | --- | --- |
| Gender |  |  |
| Male | 1651 | 1.13 (1.10, 1.17) <0.0001 |
| Female | 1348 | 1.07 (1.04, 1.11) <0.0001 |
| Age (years) |  |  |
| <65 | 1433 | 1.14 (1.10, 1.17) <0.0001 |
| ≥65 | 1566 | 1.07 (1.04, 1.10) <0.0001 |
| Ethnicity |  |  |
| Caucasian | 2072 | 1.02 (0.99, 1.05) 0.2397 |
| Black | 208 | 1.02 (0.92, 1.13) 0.6799 |
| Asian | 71 | 0.92 (0.77, 1.09) 0.3159 |
| Others | 648 | 1.00 (0.95, 1.06) 0.9252 |
| Congestive heart failure |  |  |
| No | 1935 | 1.09 (1.06, 1.12) <0.0001 |
| Yes | 1064 | 1.11 (1.07, 1.15) <0.0001 |
| Pulmonary circulatory disease |  |  |
| No | 2744 | 1.10 (1.07, 1.12) <0.0001 |
| Yes | 255 | 1.11 (1.03, 1.20) 0.0076 |
| Chronic pulmonary disease |  |  |
| No | 2189 | 1.01 (0.98, 1.04) 0.4180 |
| Yes | 810 | 1.02 (0.97, 1.07) 0.3997 |
| Renal failure |  |  |
| No | 2643 | 1.09 (1.06, 1.11) <0.0001 |
| Yes | 356 | 1.18 (1.11, 1.25) <0.0001 |
| Liver disease |  |  |
| No | 2694 | 1.10 (1.08, 1.13) <0.0001 |
| Yes | 305 | 1.06 (1.00, 1.11) 0.0469 |
| Metastatic cancer |  |  |
| No | 2808 | 1.11 (1.08, 1.13) <0.0001 |
| Yes | 191 | 1.05 (0.98, 1.12) 0.1734 |
| Mechanical ventilation |  |  |
| No | 350 | 1.13 (1.08, 1.18) <0.0001 |
| Yes | 2649 | 1.16 (1.13, 1.18) <0.0001 |

HR: indicates hazard risk; CI: confidence interval；RDW: red cell distribution width; SID30: Elixhauser Comorbidity index; APS III: Acute Physiology Score III; OASIS: Oxford Acute Severity of Illness Score; qSOFA: quick Sequential Organ Failure Assessment score; SIRS: Systemic Inflammatory Response Syndrome;

Adjusted for age, gender, ethnicity, liver disease, metastatic cancer, congestive heart failure, renal failure, APS III score, SID30, OASIS, qSOFA, SIRS and mechanical ventilation, if not stratified;

**S5: The distribution characteristics of reticulocytes according to RDW**

|  | RDW (%) | | | P-value |
| --- | --- | --- | --- | --- |
|  | ＜14.1 | ≥14.1 ＜15.7 | ≥15.7 |  |
| Reticulocyte (%)  n= 469 | 1.94 ± 1.12 | 2.43 ± 1.94 | 2.69 ± 2.23 | 0.003 |
